# A Treatise on Dislocations of the Joints

**Published:** 1844-01

**Authors:** 


Art. VII.—*A Treatise on Dislocations and Fractures of the Joints.* By Sir Astley Cooper, Bart. F. R. S., &c. *A new edition, much enlarged.* Edited by Bransby B. Cooper, F. R. S., &c. With additional observations, and a memoir of the author. Philadelphia: Lea & Blanchard. 1844. 8vo. pp. 499.

This Treatise has been long before the profession, and the new edition of it, published under the superintendence of the Committee on Publications of the Massachusetts Medical Society, embraces valuable improvements, as well in the matter of the work, as in the style in which it has been brought out. It is as tasteful a volume as the student could desire to see—on a neat, clear type, white paper, and with numerous wood engravings of capital execution. As to matter, it is an embodiment of the results of the large experience and observation of Sir Astley's long life. It is one of the best productions of the first surgeon of his age—an eminently practical treatise on subjects of the highest interest to the practitioner. No cases oftener occur in the practice of a young surgeon than those of dislocations and fractures, and none, we may add, are more frequently mismanaged. Let every young practitioner make himself familiar with the principles laid down in this work, and cases of malpractice, in casualties of the sort, will become matters of less frequent occurrence.

From the interesting memoir of the author, prefixed to this volume, we make a single short extract—it is a part of the account given of the examination of his body, after death:

"Sir Astley had requested the examination," says his biographer, "and had directed the attention of the examiners to several points of inquiry; to wit, to the marks of an umbilical, and an inguinal hernia, of each of which he had been cured; to some suspected indications of phthisis in his youth, and of which several individuals of his family had died, and an inability to sleep when lying on his left side.

"Some remains of both the hernia were discovered; and at the superior and posterior part of the right lung was a small depressed and somewhat contracted surface, about the extent of a sixpence; a section of which exposed a calcareous mass, very uneven upon its surface, and about equal to the size of a small pea."

